# Retinal Artery Occlusion and Its Impact on the Incidence of Stroke, Myocardial Infarction, and All-Cause Mortality during 12-Year Follow-Up

**DOI:** 10.3390/jcm11144076

**Published:** 2022-07-14

**Authors:** Joanna Roskal-Wałek, Paweł Wałek, Michał Biskup, Jacek Sidło, Elżbieta Cieśla, Dominik Odrobina, Jerzy Mackiewicz, Beata Wożakowska-Kapłon

**Affiliations:** 1 Collegium Medicum, Jan Kochanowski University, 25-317 Kielce, Poland; joanna.roskal.walek@wp.pl (J.R.-W.); eciesla@ujk.edu.pl (E.C.); magdale_5@hotmail.com (D.O.); bw.kaplon@poczta.onet.pl (B.W.-K.); 2 Ophthalmology Clinic, Voivodeship Regional Hospital, 25-736 Kielce, Poland; michal.biskup@onet.pl (M.B.); jaceksidlo@wp.pl (J.S.); 3 1st Clinic of Cardiology and Electrotherapy, Swietokrzyskie Cardiology Centre, 25-736 Kielce, Poland; 4 Ophthalmology Clinic Boni Fratres Lodziensis, 93-357 Łódź, Poland; 5 Department of Vitreoretinal Surgery, Medical University of Lublin, 20-079 Lublin, Poland; jerzymackiewicz@umlub.pl

**Keywords:** retinal artery occlusion, stroke, myocardial infarction, all-cause mortality

## Abstract

The aim of the study was to evaluate the incidence of ischemic stroke, myocardial infarction, and all-cause mortality in patients with retinal artery occlusion (RAO). This single-center retrospective study included 139 patients diagnosed with RAO between 2009 and 2020. The control group included 139 age- and sex-matched patients without RAO who underwent cataract surgery. The year of the surgery corresponded to the year of RAO onset. During the 12-year follow-up, patients with RAO had a shorter time to death (49.95 vs. 15.74 months; *p* = 0.043), a higher all-cause mortality rate (log-rank *p* = 0.026, and a higher rate of the composite endpoint, including ischemic stroke, myocardial infarction, and all-cause mortality (log-rank *p* = 0.024), as compared with controls. Patients with RAO younger than 75 years showed a higher risk of cerebral ischemic stroke (log-rank *p* = 0.008), all-cause mortality (log-rank *p* = 0.023), and the composite endpoint (log-rank *p* = 0.001) than controls. However, these associations were not demonstrated for patients aged 75 years or older. Our study confirms that patients with RAO have a higher risk of all-cause mortality than those without RAO. Moreover, patients with RAO who are younger than 75 years are significantly more likely to experience ischemic stroke, death, or the composite endpoint after an occlusion event, as compared with individuals without RAO.

## 1. Introduction

Retinal artery occlusion (RAO) is a visually disabling ocular vascular occlusive disorder. In addition to visual impairment, RAO is associated with a higher incidence of systemic morbidity [1,2]. Large-vessel disease and embolism from carotid artery plaques are recognized as the most common etiology of RAO, while the second most common cause is cardiac embolism [1,2]. RAO can be divided into central retinal artery occlusion (CRAO) and branch retinal artery occlusion. Importantly, not all cases of RAO are due to embolism, and there may be various other potential causes, including vasculitis [2]. RAO typically affects elderly patients burdened with numerous cardiovascular risk factors that often remain undiagnosed. The most frequently identified cardiovascular risk factors are hypertension, dyslipidemia, smoking, and diabetes [1,2,3,4,5,6,7,8].

Due to the similarities in pathogenesis and cardiovascular risk factors, an episode of RAO was considered the equivalent of a stroke. As such, it has received considerable attention, especially in the context of subsequent ischemic events [9,10,11,12,13,14,15,16,17,18,19,20,21,22,23]. To date, there have been only a few large population-based studies assessing the risk of stroke, myocardial infarction, and all-cause mortality in patients with RAO compared with controls [10,13,24,25]. Although these studies included different populations, they all demonstrated that patients with a history of RAO have a higher risk of subsequent stroke and myocardial infarction compared with controls, with the highest risk of stroke in the first two weeks after an occlusion episode [10,13,24,25].

The above results were not confirmed by single-center studies based on a direct patient evaluation [6,11,17,26]. None of those studies reported such a high risk of ischemic stroke following RAO, and some of them even questioned the findings of the previous large analyses. Considering that the clinical management of patients with acute RAO remains controversial, it seems particularly important to address these discrepancies in research [27,28,29]. An increase in the rate of stroke and hospitalizations associated with RAO was reported previously [18,30]. In the aging European population, cardiovascular disease remains one of the main causes of morbidity and mortality [31]. Therefore, it still seems reasonable to determine the risk of ischemic events after RAO, especially that to date only one study has assessed the long-term risk of stroke, myocardial infarction, and all-cause mortality in European patients with RAO as compared with controls [25].

The aim of our study was to assess the incidence of ischemic stroke, myocardial infarction, and all-cause mortality in patients with a history of RAO compared with age- and sex-matched controls with a history of cataract surgery and without ocular vascular occlusive disorders.

## 2. Materials and Methods

### 2.1. Database

The study was conducted according to the guidelines of the Declaration of Helsinki and approved by the Ethics Committee of Jan Kochanowski University in Kielce, Poland (the approval number 5/2021). Informed consent was not obtained because the data were analyzed anonymously. The patient’s waiver of consent to participate in the study was approved by the Ethics Committee. We enrolled 139 patients with RAO who were hospitalized in the Department of Ophthalmology from 2009 to 2020 due to RAO confirmed by an ophthalmologic examination. Data on hospitalization for myocardial infarction, stroke, and all-cause mortality were obtained from the Polish National Health Fund. The institution finances health services provided to insured Polish citizens and collects patient data, including data on the causes and dates of hospitalization as well as on medical procedures performed during hospital stays. The International Classification of Diseases, 10th Revision (ICD-10) system was used to encode the causes of hospitalization. Each Polish patient is identified in the healthcare system with a personal identity number of the Universal Electronic System for Registration of the Population.

### 2.2. Sample Study

All inpatients treated for RAO (ICD-10: H34.1, H34.9) at the Department of Ophthalmology between 2009 and 2020 were included in this retrospective study. The inclusion of patients with RAO in the study was described previously [21]. The control group included sex- and age-matched patients without ocular vascular occlusive disorders who were randomly selected from a total of 15,373 patients undergoing cataract surgery between 2009 and 2020 at the Department of Ophthalmology. The year of the surgery corresponded to the year of RAO onset. The final study sample included 139 patients with the diagnosis of RAO confirmed by ophthalmologic examination and 139 patients without RAO and with a history of cataract surgery.

Ophthalmologic examination after cataract surgery could exclude patients with CRAO. A thorough ophthalmologic examination of patients following cataract surgery revealed branch RAO in one patient randomly assigned to the control group. After additional tests, the patient was excluded from the control group. Each patient was followed for up to 12 years from the time of RAO diagnosis or cataract surgery. Data on concomitant diseases were obtained from the hospitalization records. Patients were classified as having hypercholesterolemia based on the diagnosis of hypercholesterolemia before admission, elevated total cholesterol (>190 mg/dL) or low-density lipoprotein (LDL) cholesterol levels on admission, or the use of lipid-lowering drugs at the time of an RAO episode or cataract surgery. Patients with ischemic heart disease were identified based on a history of myocardial infarction, percutaneous coronary intervention, coronary artery bypass grafting, or a diagnosis of ischemic heart disease by a cardiologist. The characteristics of the medications taken represent the pharmacotherapy that patients were taking on admission to the hospital for RAO or cataract surgery. At discharge after an episode of RAO, patients were administered acetylsalicylic acid or an oral anticoagulant, as indicated, if those medications were not previously taken for atrial fibrillation (AF).

The first day of hospitalization for ischemic stroke or myocardial infarction was considered the date of that event in both groups. When several hospitalizations were recorded for a particular patient, the date of the first stay was used in the analysis. ICD-10 codes I63, I63.0, I63.1, I63.2, I63.3, I63.4, I63.5, I63.8, and I63.9 were considered to indicate stroke, while I21, I21.0, I21.2, I21.3, I21.4, and I21.9, myocardial infarction. National Health Fund data were used to determine the date of death from any cause. Each patient was followed for up to 12 years from the time of RAO diagnosis or cataract surgery.

### 2.3. Statistical Analysis

The results were presented with means ± standard deviations (SDs) or counts and percentages. Student’s *t*-test (for normally distributed variables), the Mann–Whitney test, and the Chi-squared test (for non-normally distributed variables) were used to compare the demographic and clinical data of the study groups. The baseline characteristics of patients with RAO and cataract surgery were recorded at the time of the RAO episode or cataract surgery. Times to event were presented as medians with interquartile ranges and compared using the Mann–Whitney test. The composite endpoint was defined as the occurrence of ischemic stroke, myocardial infarction, or all-cause mortality. The percentage of patients free from stroke, myocardial infarction, all-cause mortality, and composite endpoint were estimated using the Kaplan–Meier method. The log-rank test was used to compare the Kaplan–Meier curves between patients with RAO and controls. The Kaplan–Meier analysis was performed for the entire study population and separately for patients younger than 75 years and those aged 75 years or older.

The statistical significance threshold of *p* < 0.05 was adopted. The STATISTICA 13.3 software (TIBCO Software Inc., Tulsa, OK, USA) was used for the analysis.

## 3. Results

The characteristics of the study groups are presented in Table 1. Patients with a history of RAO were more likely than controls to have hypertension (85.61% vs. 64.75%; *p* < 0.001), hypercholesterolemia (73.38% vs. 27.34%; *p* < 0.001), ischemic heart disease (41.73% vs. 30.22%; *p* = 0.046), and heart failure (20.14% vs. 10.07%; *p* = 0.019). In addition, they were more likely to have a history of myocardial infarction (24.46% vs. 8.63%; *p* < 0.001) and ischemic stroke (12.23% vs. 4.32%; *p* = 0.017) and were more often smokers (25.18% vs. 8.63%; *p* < 0.001) (Table 1).

In patients with RAO, a similar incidence of ischemic stroke before and after RAO was observed (12.23% vs. 10.07%; *p* = 0.567), while myocardial infarction occurred more often before RAO (24.46% vs. 2.16%; *p* < 0.001). The incidence of cardiovascular disease before RAO resulted in a more frequent use of acetylsalicylic acid as secondary prevention of cardiovascular events among RAO patients than among controls (23.02% vs. 10.79%; *p* = 0.007). However, there were no differences in the incidence of diabetes (20.14% vs. 29.5%; *p* = 0.071) or AF (15.11% vs. 9.35%; *p* = 0.143) between patients with RAO and controls.

The analysis showed no differences in the incidence of ischemic stroke (10.07% vs. 5.04%; *p* = 0.112), myocardial infarction (2.16% vs. 1.44%; *p* = 0.652), all-cause mortality (25.9% vs. 17.27%, *p* = 0.080), or the composite endpoint (30.22% vs. 20.86%; *p* = 0.074) after an RAO episode or cataract surgery for the entire study group during the 12 years of follow-up. Patients with RAO had a shorter time to all-cause mortality than controls (39.6 vs. 69.72 months; *p* = 0.043) during the follow-up.

**Figure 1:**
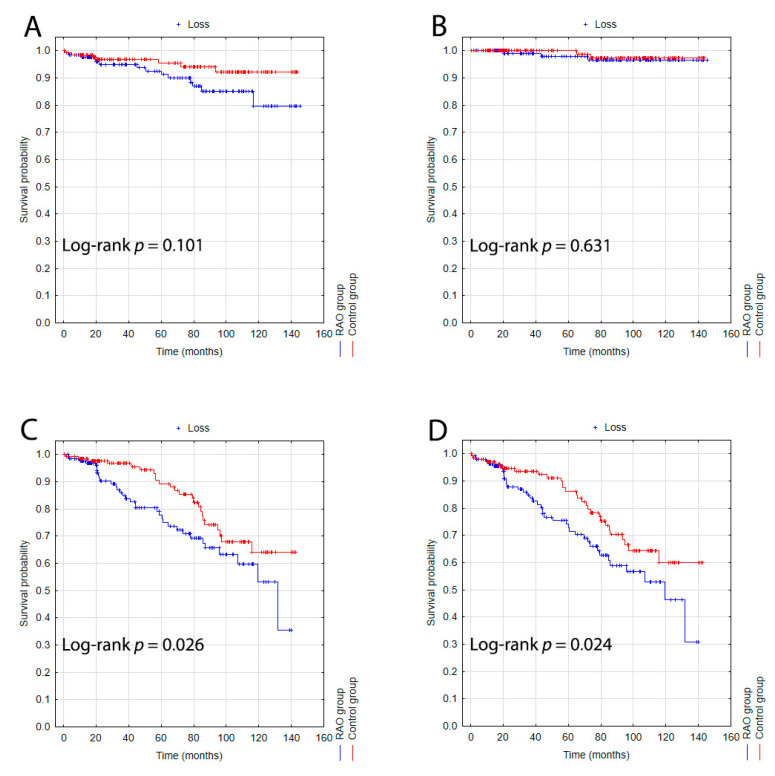
Kaplan–Meier survival probability curves for the whole study population (RAO, blue line; control group, red line) for ischemic stroke (**A**), myocardial infarction (**B**), all-cause mortality (**C**), and the composite endpoint (**D**).

The Kaplan–Meier curves for the RAO and control groups are shown in Figure 1. In the Kaplan–Meier analysis for the incidence of stroke in the RAO and control groups, despite the apparent divergence of the curves, the log-rank test did not show statistically significant differences between groups for the 12-year follow-up. In addition, the analysis revealed no significant differences in the incidence of myocardial infarction between the two groups. However, it should be noted that we recorded three and two myocardial infarctions in the RAO and control groups, respectively. The analysis for all-cause mortality showed that patients with a history of RAO had a significantly higher mortality rate than controls. The curves suddenly diverged at 20 months of follow-up, and then appeared to converge again around month 80. Finally, the Kaplan–Meier analysis for the composite endpoint showed a significantly higher rate of events in the RAO group than in the control group. For the composite endpoint, the curves suddenly diverged around month 20 of follow-up.

**Figure 2:**
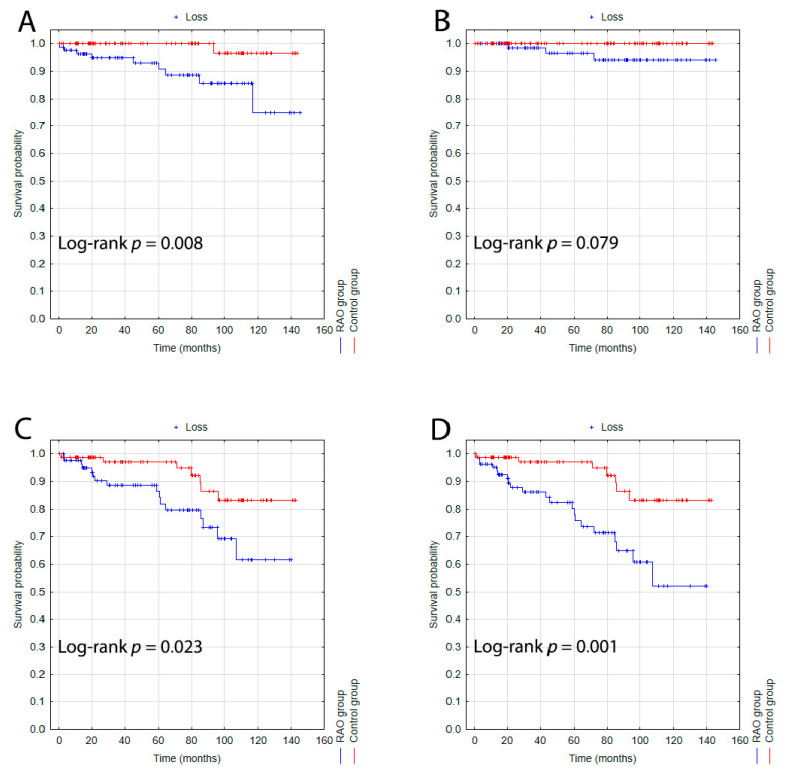
Kaplan–Meier survival probability curves for patients younger than 75 years old in the whole study group (RAO, blue line; control group, red line) for ischemic stroke (**A**), myocardial infarction (**B**), all-cause mortality (**C**), and composite endpoint (**D**).

Due to the advanced age of the study population, the shorter time to all-cause mortality in the RAO group, and the convergence of the Kaplan–Meier curves along with follow-up, the Kaplan–Meier analysis was performed according to age at the time of the RAO event or cataract surgery. Among participants younger than 75 years, stroke, all-cause mortality, and the composite endpoint were reported more often for patients with RAO than for controls (Figure 2).

**Figure 3:**
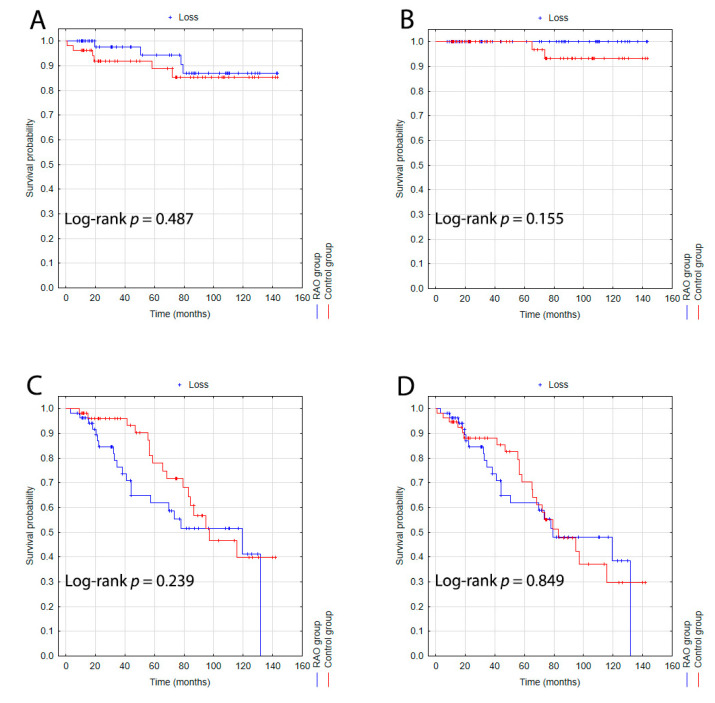
Kaplan–Meier survival probability curves for patients aged 75 years or older in the whole study population (RAO, blue line; control group, red line) for ischemic stroke (**A**), myocardial infarction (**B**), all-cause mortality (**C**), and composite endpoint (**D**).

Importantly, in the control group, among patients younger than 75 years, the first ischemic stroke was recorded around month 90 of follow-up, and there was no sudden increase in the rate of all-cause mortality until around month 70. In patients aged 75 years or older, there were no significant differences in the incidence of stroke, myocardial infarction, all-cause mortality, or composite endpoint between the RAO and control groups (Figure 3).

The person-years and incidence rates of ischemic stroke, myocardial infarction, death, and composite endpoint per 1000 person-years in patients with RAO and controls are presented in Table 2.

## 4. Discussion

In our study, we found that patients who experienced an episode of RAO have a worse prognosis in terms of death and the composite endpoint of ischemic stroke, myocardial infarction, and all-cause mortality compared with the control group without ocular vascular occlusive disorders. Despite the divergence of the curves in the Kaplan–Meier analysis with regard to the incidence of ischemic stroke after RAO, the observation was statistically insignificant in the log-rank test. This may be due to the effect of group size, patient age, or study duration. We found that among participants younger than 75 years, patients with RAO had a worse prognosis in terms of ischemic stroke, death, and the composite endpoint than controls. Such an association was not observed for participants aged 75 years or older. Patients with a history of RAO had a shorter time to death than those in the control group, which is in line with other studies. Lorenzten et al. reported that the lifetime of patients with CRAO is reduced by 10 years as compared with healthy controls [32]. The analysis of two large population studies showed that the mortality of patients who developed RAO was almost twice as high as that of patients without RAO (56% vs. 30%) over a 10-year follow-up [33]. In a study on the European population, Hankey et al. reported all-cause mortality in 29.59% of participants during the 10-year follow-up, which is in line with our results [4]. Of note, however, the Hankey study did not include a control group [4]. In another European study, Vestergaard et al. reported all-cause mortality within 1 year of RAO onset in 6.06% of patients, which was significantly higher than in the control group [25].

In our study, the comparison of the number of deaths between patients with RAO and controls did not show significant differences during the 12-year follow-up. However, in the Kaplan–Meier analysis, patients with RAO had a worse prognosis than the control group. For the incidence of all-cause mortality, the analysis showed a sudden divergence of the curves around month 20, but then the curves converged again around month 80 or 90. A similar tendency, although less pronounced, was shown for the composite endpoint, which also included all-cause mortality. In our opinion, the convergence of the Kaplan–Meier curves may have been caused by the advanced age of the study group, the shorter time to death in the RAO population, and the long follow-up. Therefore, we performed a separate Kaplan–Meier analysis according to age, with a cut-off value of 75 years following the World Health Organization’s definition of senile age. The analysis showed that RAO patients under the age of 75 years had worse prognosis than controls, which was not observed for older patients. In our opinion, the comparable risk of ischemic stroke, myocardial infarction, all-cause mortality, and the composite endpoint in older individuals was due to their baseline age-related risk of these events.

The vast majority of RAO affects elderly patients, in which traditional cardiovascular risk factors have important underlying role in the onset of the disease. The presence of numerous cardiovascular risk factors in patients with RAO has been reported by multiple studies [1,2,3,4,5,6,7,8,10,13,15,18,20,21,24,25]. In a study by Callizo et al., 78% of patients had at least one undiagnosed cardiovascular risk factor at the time of CRAO [3]. In our research, we found that RAO patients were more likely to have hypertension, hypercholesterolemia, ischemic heart disease, heart failure, and a history of smoking as compared with controls. This is consistent with the results of other studies comparing RAO patients and controls [2,7,13,20]. Similar to many other studies we have also found hypertension as one of the most frequent risk factors in patients with RAO [2,3,4,5,6,7,18,23,30]. In addition to traditional cardiovascular risk factors, hypercoagulable states are also mentioned among the risk factors for RAO, especially in young adults. The possible significance of thrombophilia in RAO as well as in ischemic stroke remains controversial [34,35,36]. Recently published systematic reviews and meta-analysis revealed that patients with retinal vascular occlusion showed similar prevalence of inherited and acquired thrombophilias compared with healthy subjects. Nevertheless, it should be noted that most of the patients included in the studies of this meta-analysis were predominantly elderly and this fact may have an impact on the overall incidence of thrombophilia tested [35]. However, in a study by Ward et al. that assessed the presence of risk factors for RAO in young adults, hypercoagulable states were the most commonly diagnosed risk factors [36].

Apart from the presence of numerous cardiovascular risk factors, patients with RAO more often have a positive history of cerebrovascular accident than controls [2]. In our study, 12.23% of patients had a history of ischemic stroke before RAO. This is in line with the results by Callizo et al., who reported a history of ischemic stroke in 11.68% of patients [3]. According to Xiao et al., a history of ischemic stroke is associated with an increased risk of RAO, which indirectly suggests that RAO is part of the stroke continuum and thus deserves an in-depth study [37].

Because RAO is a rare condition, data on risk assessment for stroke and myocardial infarction after RAO are derived mainly from large population studies based on the analysis of ICD-10 codes [10,13,20,24,25]. Studies on the Asian population showed a higher risk of stroke after RAO, particularly in the period immediately after the event. Chang et al. reported that 19.61% of patients with RAO experienced stroke during the 3-year follow-up, as compared with 10.05% of controls. The highest incidence rate ratio of stroke was 9.46 within a month after RAO, followed by 5.57 at 1–6 months and 2.16 at 3 years [24]. A significantly higher risk of stroke in patients with a history of RAO compared with the control group was also demonstrated by Rim et al. (hazard ratio, 1.78; 95% CI, 1.32–2.41) [20]. The study had a follow-up duration of 10 years, but the exact time relationship for the incidence of stroke after RAO was not reported. Chang et al. reported a higher risk of acute coronary syndrome (ACS) in patients after RAO compared with controls who were matched for age, sex, and comorbidities, which strengthened the significance of the findings. After adjustment for potential confounders, the risk of ACS was 1.72-fold higher (95% CI, 1.20–2.47) in RAO patients than in controls [10]. Park et al. investigated the risk of stroke and myocardial infarction around the time of CRAO [19]. They reported that the risk of ischemic stroke was closely associated with the time of RAO onset and was the highest in the first week after RAO. No such association was found for hemorrhagic stroke or myocardial infarction [19]. The increased risk of stroke around the time of CRAO was confirmed also by studies on the Caucasian population [13,22]. French et al. showed that patients with CRAO had a significantly higher risk of ischemic stroke after CRAO than patients in the control group. The highest risk was recorded in the second week after CRAO [13]. This is contrast to our study, in which we did not observe an increased risk of ischemic stroke in the first weeks after RAO.

In a recent study, Vestergaard et al. assessed the risk of stroke, myocardial infarction, and all-cause mortality after RAO in the European population [25]. Similar to previous studies based on large databases, the authors showed a significantly higher risk of stroke and myocardial infraction after RAO compared with controls. This study also showed that patients after RAO have significantly higher risk of all-cause mortality after RAO compared with controls [10,13,20,24,25]. In contrast to the studies by Chang et al. and French et al., the risk was assessed after adjustment for confounders, such as diabetes mellitus, hypertension, heart failure, chronic kidney disease, cancer, ischemic heart disease, stroke, and AF [13,24]. The highest risk of stroke was noted from days 3 to 14 after RAO with an adjusted risk ratio of 50.71 (95% CI, 41.55–61.87) [25]. Like Chang et al., Vestergaard et al. reported a more frequent occurrence of myocardial infarction in patients after RAO compared with the control group [10,25]. The time relationship between RAO and myocardial infarction was noticeable in this study, which is in contrast to the results of Park et al. RAO was associated with an increased risk of myocardial infarction, with the highest risk from days 14 to 90 after RAO (risk ratio, 1.98; 95% CI, 1.25–3.15) [19,25]. In our study, only three patients (2.16%) had myocardial infarction during the 12-year follow-up, while Laczynski et al. reported this event in 3.6% of cases during a comparable follow-up [6]. Interestingly, in the long-term follow-up study by Hankey et al., the rate of myocardial infarction was higher than that of stroke and coronary events were the main cause of death [4]. This is in contrast to our results and those of Vestergaard et al. showing that stroke was more frequent than myocardial infarction after RAO [25]. This finding is even more surprising considering that in our study there was a high percentage of RAO patients diagnosed with ischemic heart disease, including myocardial infarction. One possible explanation is that the Hankey study was conducted over 30 years ago, and today there are much better diagnostic methods available, along with an improved secondary prevention measures for cardiovascular risk factors [4].

Vestergaard et al. reported that 5.89% of strokes occurred within 1 year of RAO, but the overall number of strokes during the follow-up was not provided [25]. In our study, 10.07% of patients experienced stroke during the 12-year follow-up, which is similar to the findings by Hankey et al. who reported a rate of 10.2% for the European population over 10 years of follow-up (although without a comparison with the control group) [4]. On the other hand, Rim et al. reported a rate of 15% among Asian patients with RAO during the 10-year follow-up, which is significantly higher than in our study [20]. Importantly, however, our studies differed not only in the population but also methodology. Rim et al. assessed also hemorrhagic and unspecified stroke [20]. Nevertheless, compared with the studies by Rim et al., Vestergaard et al., or Chang et al., we did not find a significant difference in the incidence of stroke after RAO compared with the control group [20,24,25]. This may be related to the small sample size or to the advanced age of the study group, because the risk of ischemic events is higher with increasing age and higher cardiovascular burden. Nevertheless, after dividing the study group by age (<75 years vs. ≥75 years), significant differences were found both for stroke and for all-cause mortality. This is an important relationship indicating that the occurrence of RAO may have a strong impact on the risk of stroke in younger age groups, as reported also by other studies [19,20,24].

The lower rate of stroke after RAO may be also explained by the fact that many of our patients suffered from stroke or myocardial infarction before RAO onset and they received antiplatelet therapy as secondary prevention. It is noteworthy that RAO occurred despite this treatment. Interestingly, recent studies indicated that antiplatelet and anticoagulant medications do not reduce the risk of stroke following RAO [25,26]. One possible explanation is that RAO and subsequent stroke are caused by a different type of emboli than those causing classic stroke. This hypothesis was proposed by some authors to explain why thrombolytic therapy does not work in the acute management of RAO. Indeed, the majority of retinal emboli were shown to be made of cholesterol or calcified material [25]. It can be assumed that antiplatelet and anticoagulant drugs most likely protect against macroangiopathic complications by inhibiting large thrombus formation, but they do not protect against microangiopathic complications, such as RAO, which can be caused by cholesterol or calcified material.

Another possible reason for the lower rate of stroke in our study may be the fact that it was designed as a single-center registry. Recently, Laczynski et al. undermined the results of large population-based studies by reporting a much lower incidence of stroke after RAO [6]. Other single-center registry studies also showed a much lower rate of stroke after RAO compared with large database studies [17,37]. Although the risk of stroke in single-center studies was much lower than that reported by large population studies, it is important to note that in the study by Laczynski et al., four of the five strokes occurred simultaneously with RAO, and they were minor strokes [6]. Similarly, in the study by Hoyer et al., in 25 of the 77 patients with RAO, acute ischemic lesions were observed on magnetic resonance imaging (MRI); four patients had focal symptoms, two patients had TIAs, one patient had severe stroke, and the remaining lesions were clinically silent [30]. Even in the study by Hankey et al., two-thirds of strokes after RAO were non-disabling [4]. Nevertheless, the occurrence of minor stroke and TIA has significant clinical implications. A recent multicenter study showed that in patients with minor stroke or TIA, the risk of early stroke recurrence or neurological deterioration was only 2% in the absence of infarction on neuroimaging or the large artery disease stroke subtype; however, in cases with large artery disease stroke subtype with infarction on neuroimaging, the risk was approximately 30% [38]. The EXPRESS [39] and SOS-TIA [40] studies both demonstrated that prompt evaluation and immediate treatment after TIA and minor stroke greatly reduced the risk of recurrent stroke (by 80%).

Studies with diffusion-weighted magnetic resonance imaging within 7 days of RAO (i.e., the period indicated in other studies as associated with the highest frequency of stroke) reported a high percentage of acute ischemic lesions in the central nervous system, most of which are silent brain infarctions (SBIs) [12,14,16]. It cannot be excluded that recent improvements in the recognition of these lesions after RAO are responsible for the higher percentage of strokes reported after RAO in large database studies. As in our study patients did not undergo MRI, we cannot comment on the percentage of such events after RAO. It is also certain that owing to the lack of MRI, SBIs were not listed among ICD codes. Moreover, such a high percentage of SBIs and the time relationship with the occurrence of RAO may indicate that RAO is closest to this type of stroke. The relationship between SBI and RAO provides valuable information. First, these lesions are not benign, as originally believed, and they are associated with subtle neurological deficits, cognitive dysfunction, psychiatric disorders, clinically apparent stroke, and early mortality [41]. Second, in studies that found SBI on MRI in patients with RAO, the etiology of RAO (carotid atherosclerosis followed by cardioembolism) was significantly more often determined than in patients with RAO without SBI. The determination of stroke etiology is of key importance for decision making regarding the treatment method, the timing of diagnostic tests, and prevention [42]. In a study by Hong et al., large artery atherosclerosis was the most common etiology of RAO. Patients with RAO and large artery atherosclerosis had a four-fold greater risk of ischemic events than those with RAO but without atherosclerosis [5].

Further research into the relationship between RAO and SBI as well as leukoaraiosis, which are part of the spectrum of cerebral small vessel disease, may shed new light on the etiopathogenesis of these events. Recent studies indicated a significant overlap of large artery atherosclerosis and small vessel disease in the pathogenesis of RAO, and this has implications for the assessment of overall cerebrovascular risk, which should take into account both macroangiopathic and microangiopathic lesions [43,44].

### 4.1. Strenghts

Only patients with the diagnosis of RAO confirmed by our research group were included in this study, thus excluding misdiagnoses. We reviewed the medical records of all patients to confirm RAO, and we excluded two patients who were suspected of Horton’s disease and Susac syndrome. The study population is homogeneous in terms of ethnicity because only European patients were included in this study. This is important, as differences in the risk of ischemic stroke and RAO between ethnic groups have been reported. The follow-up lasted 12 years, which is one of the longest follow-up durations in the RAO setting. Instead of monitoring only one selected cardiovascular event, our patients were monitored for the incidence of ischemic stroke, myocardial infarction, and all-cause mortality, which allows for a broad assessment of cardiovascular risk after RAO. Finally, for our study, we randomly selected a control group matched for sex, age, and the year of the RAO event/cataract surgery. Each control patient was assessed for ocular vascular occlusive disorders, and any patients suspected of RAO were excluded from the control group.

### 4.2. Limitations

Due to the lack of detailed information on the cause of death, especially sudden death, we used all-cause mortality for the analysis. In some cases, death may have been caused by undiagnosed ischemic stroke or myocardial infarction. This might have reduced the percentage of accumulated cardiovascular events, including ischemic stroke or fatal myocardial infarction. Therefore, we decided to use a composite endpoint in the analysis. Unlike RAO diagnosis, the diagnoses of ischemic stroke and myocardial infarction could not be verified by our group because the dates of these events were derived from the National Health Fund data using ICD-10 codes. Since health insurance provided by the National Health Fund is primarily used in Poland, a bias resulting from limited reporting of events outside Poland may have occurred. However, the study was conducted in the region of central Poland, and the risk of such a bias can be considered very small. Because differences in the incidence of ischemic stroke and RAO have been reported in different ethnic groups, our results should not be generalized to other populations.

The sample was relatively small because RAO is a relatively rare disease, diagnosed and treated in ophthalmology departments. In addition, this was a single-center study. This could have influenced the borderline statistical significance in the analysis of some of the variables. Due to the small size of the study group and the burden of cardiovascular disease, it was difficult to match the control group in terms of these parameters, leading to the control group being matched only for age, sex, and the year of RAO. Adjustment only for these variables precluded the assessment of the risk of cardiovascular events that would be affected only by an RAO event. Due to the cardiovascular burden in the RAO group, we can assume that the more frequent cardiovascular events and deaths in this group of patients are due to the general risk resulting from these diseases and not only due to the previous episode of RAO. Patients in the control group did not undergo carotid Doppler ultrasound examinations, so we could not compare these parameters between the RAO and control groups, despite the fact that one of the main sources of embolic material in patients with RAO was atherosclerotic plaques located in the carotid arteries. We did not perform tests for hypercoagulability states in our population.

## 5. Conclusions

Patients with RAO are more likely to suffer from cardiovascular disease than control patients matched for sex, age, and the year of RAO event/cataract surgery, which translates into a significantly shorter life expectancy of patients after RAO. Patients with a history of RAO are at a higher risk of all-cause mortality and a composite endpoint of ischemic stroke, myocardial infarction, and all-cause mortality. The population of patients under the age of 75 with a history of RAO is more likely to experience ischemic stroke, all-cause mortality, or a composite endpoint after an RAO event than patients in the control group, which was not observed in the population of patients aged 75 years or older. In the present study, we did not observe a sudden increase in the incidence of ischemic stroke or myocardial infarction in the first weeks after an RAO event, which is in contrast to previous reports. In our population, we noted a rapid increase in the number of events approximately 20 months after an episode of RAO. Due to the higher risk of ischemic stroke, all-cause mortality, and the composite endpoint after RAO, these patients should be promptly diagnosed in terms of the etiology of RAO and related cardiovascular diseases to start treatment, which may affect their long-term prognosis. A multicenter randomized study should be designed to assess the efficacy of antiplatelet or anticoagulant treatment in patients after RAO. Considering that the main source of embolic material in patients in RAO is large artery atherosclerosis, it will be also interesting to assess the efficacy of lipid-lowering treatment in RAO patients.

## Figures and Tables

**Table 1:** Characteristics of the study groups.

	Patients with RAO *n* = 139	Control Group *n* = 139	*p*-Value
Sex (female), *n* (%)	51 (36.69)	51 (36.69)	1.000
Age, years (SD)	70.09 (11.13)	70.17 (11.49)	0.953
Age <50 years, *n* (%)	7 (5.04)	7 (5.04)	1.000
Age 50–59 years, *n* (%)	13 (9.35)	14 (10.07)	0.840
Age 60–69 years, *n* (%)	44 (31.65)	43 (30.94)	0.897
Age 70–79 years, *n* (%)	46 (33.09)	48 (34.53)	0.780
Age ≥80 years, *n* (%)	29 (2.86)	27 (19.42)	0.765
Hypertension, *n* (%)	119 (85.61)	90 (64.75)	<0.001
Hypercholesterolemia, *n* (%)	102 (73.38)	38 (27.34)	<0.001
Coronary artery disease, *n* (%)	58 (41.73)	42 (30.22)	0.046
Heart failure, *n* (%)	28 (20.14)	14 (10.07)	0.019
Myocardial infarction before RAO, *n* (%)	34 (24.46)	12 (8.63)	<0.001
Ischemic stroke before RAO, *n* (%)	17 (12.23)	6 (4.32)	0.017
Hemorrhagic stroke before RAO, *n* (%)	3 (2.16)	0 (0.0)	0.082
Smoking, *n* (%)	35 (25.18)	12 (8.63)	<0.001
Acetylsalicylic acid, *n* (%)	32 (23.02)	15 (10.79)	0.007
Diabetes mellitus, *n* (%)	28 (20.14)	41 (29.50)	0.071
Atrial fibrillation, *n* (%)	21 (15.11)	13 (9.35)	0.143
Permanent atrial fibrillation, *n* (%)	11 (7.91)	9 (6.47)	0.642
Paroxysmal atrial fibrillation, *n* (%)	10 (7.19)	4 (2.88)	0.100
Oral anticoagulants, *n* (%)	17 (12.23)	12 (8.63)	0.327
Vitamin K antagonists, *n* (%)	13 (9.35)	6 (4.32)	0.096
Non-vitamin K antagonist oral anticoagulants, *n* (%)	4 (2.88)	6 (4.32)	0.519
Ischemic stroke after RAO/cataract surgery, *n* (%)	14 (10.07)	7 (5.04)	0.112
Myocardial infarction after RAO/cataract surgery, *n* (%)	3 (2.16)	2 (1.44)	0.652
Death after RAO/cataract surgery, *n* (%)	36 (25.90)	24 (17.27)	0.080
Composite endpoint after RAO/cataract surgery, *n* (%)	42 (30.22)	29 (20.86)	0.074
Median time to stroke, months (IQR)	47.87 (60.83)	19.23 (66.77)	0.502
Median time to myocardial infarction, months (IQR)	43.23 *	69.38 *	0.248
Median time to death, months (IQR)	39.60 (49.95)	69.72 (15.74)	0.043
Median time to composite endpoint, months (IQR)	42.15 (50.83)	65.03 (58.38)	0.198

IQR, interquartile range; RAO, retinal artery occlusion; SD, standard deviation. * The IQR cannot be calculated because of an insufficient number of events.

**Table 2:** Persons-years and incidence rates for the analyzed events.

Event		RAO Group	Control Group	*p*-Value
ischemic stroke	PY	723.1	749.9	
	IR (95% CI)	17.98 (9.56–30.75)	9.33 (3.74–19.23)	0.163
myocardial infarction	PY	755.1	765.1	
	IR (95% CI)	3.97 (0.8–11.61)	2.61 (0.29–9.44)	0.677
death	PY	622.3	720.1	
	IR (95% CI)	57.9 (40.53–80.13)	33.3 (21.35–49.6)	0.035
composite endpoint	PY	600.1	692.7	
	IR (95% CI)	70 (50.45–94.62)	41.9 (28.02–60.1)	0.032

CI, confidence interval; IR, incidence rate per 1000 person-years; PY, persons-years; RAO, retinal artery occlusion.

## Data Availability

The data underlying this article will be shared on reasonable request to the corresponding author.
